# Supplementary material for: A Tree‐Based Model for Addressing Sparsity and Taxa Covariance in Microbiome Compositional Count Data
Source: Stat Med. 2026 May 8;45:e70584. doi: 10.1002/sim.70584 (PMC13155195; doi:10.1002/sim.70584)
Supplement: Supplementary file 1 — Table S1. PJAPs of the dietary variables. Table S2. Nodes with significant association with breastfeeding reported by our LTN‐based mixed‐effects model with posterior expected FDR ≤0.05. The node labels are the same as in Figure S7. Figure S1. Runtime of 10 Gibbs iterations versus number of OTUs K. Figure S2. MSE of the estimated mean (μ) and marginal correlations on the original tree, averaged across all nodes. The MSE is calculated based on 100 replicates. Figure S3. Estimated μ across nodes with λ=10. The boxplots are generated based on 100 replicates. The nodes are ordered by their depth in 𝒯1. Figure S4. Estimated marginal correlations across nodes with λ=10. The left panel shows the shallowest ten nodes in 𝒯1 while the right shows the deepest ten nodes. The boxplots are generated based on 100 replicates. The nodes are labeled by their depth in 𝒯1. Figure S5. PMAPs of the introduction of solid food. The significant OTUs reported by MaAsLin2 are marked in the same way as Figure 14. Figure S6. PMAPs of the introduction of soy products. The chain of three significant nodes and their descendant OTU 4439360 are marked in the figure. The posterior mean of α at these three nodes (from top to bottom) are 1.22, −3.12, and 3.19 respectively. The significant nodes reported by our LTN‐based mixed‐effects model and the significant OTUs reported by MaAsLin2 are marked in the same way as Figure 14. Figure S7. PMAPs of cessation of breastfeeding. The significant OTUs reported by MaAsLin2 are marked in the same way as Figure 14. Figure S8. PMAPs for rye (m=0.05). Figure S9. PMAPs for buckwheat and millet (m=0.05). Figure S10. PMAPs for eggs (m=0.05). Figure S11. PMAPs for fish (m=0.05). Figure S12. PMAPs for barley (m=0.05). [file SIM-45-0-s001.pdf]

Supplementary materials for "A tree-based model for addressing sparsity and taxa covariance in microbiome compositional count data"

Zhuoqun Wang
Duke University
Durham, NC 27708

Jialiang Mao
LinkedIn Corporation
Sunnyvale, CA 94085

Li Ma*
University of Chicago
Chicago, IL 60637

A Technical details for Bayesian computation with LTN

A.1 Pólya Gamma augmentation for LTN

In this section we describe the Pólya Gamma augmentation scheme for the proposed LTN method. The binomial sampling model is

$$y(A_l)|\psi(A), y(A)) \overset{\text{ind}}{\sim} \text{Binomial}\left(y(A), \frac{e^{\psi(A)}}{e^{\psi(A)}+1}\right) \qquad \text{for all } A \in \mathcal{I}$$

Following Polson et al. (2013), we can write the binomial likelihood for a sample at an interior node A as

$$p(y(A_l)|y(A), \psi(A)) \propto \frac{\left(e^{\psi(A)}\right)^{y(A_l)}}{\left(1+e^{\psi(A)}\right)^{y(A)}} = 2^{-y(A)} e^{\kappa(A)\psi(A)} \int_0^\infty e^{-w\psi(A)^2/2} f(w)dw,$$

where $\kappa(A) = y(A_l) - y(A)/2$ and

$$f(w) = \frac{2^{y(A)-1}}{\Gamma(y(A))} \sum_{n=0}^{\infty} (-1)^n \frac{\Gamma(n+y(A))}{\Gamma(n+1)} \frac{(2n+y(A))}{\sqrt{2\pi w^3}} e^{-\frac{(2n+y(A))^2}{8w}}$$

*Email: li.ma@uchicago.edu.

is the probability density function of the Pólya-Gamma distribution $\text{PG}(y(A), 0)$. We can accordingly introduce an auxiliary variable $w(A)$ that is independent of $y(A_l)$ given $y(A)$ and $\psi(A)$, with

$$p(w(A)|y(A), \psi(A)) \propto e^{-w(A)\psi(A)^2/2} f(w(A)).$$

In other words, we add the auxiliary variable $w(A)$ into the LTN model in the main-text generative formulation with

$$w(A)|y(A), \psi(A) \sim \text{PG}(y(A), \psi(A)).$$

The joint conditional distribution for $w(A)$ and $y(A_l)$ given $y(A)$ and $\psi(A)$ is then

$$p(w(A), y(A_l)|y(A), \psi(A)) \propto 2^{-y(A)} e^{\kappa(A)\psi(A) - w(A)\psi(A)^2/2} f(w(A)),$$

which is a log-quadratic function of $\psi(A)$, and thus is conjugate to the multivariate Gaussian likelihood of $\boldsymbol{\psi} = \{\psi(A) : A \in \mathcal{I}\}$.

A.2 Gibbs sampler for the mixed-effects model

For notational simplicity, we rewrite the model in the following form:

$$\underset{n\times d}{\boldsymbol{\Psi}} = \underset{n\times 1}{\boldsymbol{s}}\underset{1\times d}{\boldsymbol{\alpha}} + \underset{n\times q}{\boldsymbol{Z}}\underset{q\times d}{\boldsymbol{\beta}} + \underset{n\times G}{\boldsymbol{H}}\underset{G\times d}{\boldsymbol{\Gamma}} + \underset{n\times d}{\boldsymbol{\epsilon}},$$

where $H_{ij} = I(g_i = j)$. We denote the Pólya-Gamma auxiliary variable of node A and sample i with $w_i(A)$, and let $\Sigma_\epsilon = \text{diag}(\sigma_\epsilon^2(A) : A \in \mathcal{I})$, $\phi_\epsilon(A) = \sigma_\epsilon^{-2}(A) \sim \text{Gamma}(c_0, d_0)$.

The sampler cycles through the following steps:

- Sample $\boldsymbol{\beta}$ from

$$\boldsymbol{\beta}|- \sim N_{q\times d}((\boldsymbol{Z}^T\boldsymbol{Z})^{-1}\boldsymbol{Z}^T(\boldsymbol{\Psi} - \boldsymbol{H}\boldsymbol{\Gamma} - \boldsymbol{s}\boldsymbol{\alpha})(\boldsymbol{\Sigma}_\epsilon^{-1} + \boldsymbol{I}/(cn))^{-1}\boldsymbol{\Sigma}_\epsilon^{-1},$$
$$(\boldsymbol{\Sigma}_\epsilon^{-1} + \boldsymbol{I}/(cn))^{-1} \otimes (\boldsymbol{Z}^T\boldsymbol{Z})^{-1}).$$

- For each $A \in \mathcal{I}$, sample $\alpha(A)$ from

$$\alpha(A)|- \sim (1-\pi'(A))\delta_0(\alpha(A)) + \pi'(A)(1-\delta_0(\alpha(A)))N(b(A)s_\alpha^2(A), s_\alpha^2(A)),$$

  where

$$\pi'(A) = \frac{\pi(A)s_\alpha(A)\phi_\alpha^{1/2}\exp\{b^2(A)s_\alpha^2(A)/2\}}{1-\pi(A)+\pi(A)s_\alpha(A)\phi_\alpha^{1/2}\exp\{b^2(A)s_\alpha^2(A)/2\}},$$

$$s_\alpha(A) = (\phi_\alpha + \sum_{i=1}^N s_i^2\phi_\epsilon(A))^{-1/2}, b(A) = \phi_\epsilon(A)\sum_{i=1}^N (s_i(\psi_i(A) - \gamma_i(A) - \boldsymbol{z}_i^T\boldsymbol{\beta}(A))).$$

- For each $A \in \mathcal{I}$, sample $\pi(A)$ from

$$\pi(A)|- \sim \mathrm{Beta}(m + 1 - I(\alpha(A) = 0), 1 - m + I(\alpha(A) = 0)).$$

- For $l = 1, \cdots, G$, let

$$n_l = \sum_{i=1}^n I(g_i = l), \boldsymbol{C}_l = (n_l\boldsymbol{\Sigma}_\epsilon^{-1} + \boldsymbol{\Omega})^{-1}, \boldsymbol{m}_l = \boldsymbol{C}_l(\boldsymbol{\Sigma}_\epsilon^{-1}\sum_{i:g_i=l}(\boldsymbol{\psi}_i - \boldsymbol{\beta}^T\boldsymbol{z}_i - s_i\boldsymbol{\alpha}^T)),$$

  and sample $\boldsymbol{\gamma}_l$ from

$$\boldsymbol{\gamma}_l|- \sim N(\boldsymbol{m}_l, \boldsymbol{C}_l).$$

- For each $A \in \mathcal{I}$, sample $\phi_\epsilon(A)$ from

$$\phi_\epsilon(A)|- \sim \mathrm{Gamma}(c_0 + n/2, d_0 + \sum_{i=1}^n \epsilon_i^2(A)/2)$$

- For $i = 1, \cdots, n$, let

$$\boldsymbol{C}_i = (\mathrm{diag}(\boldsymbol{w}_i) + \boldsymbol{\Sigma}_\epsilon^{-1})^{-1}, \boldsymbol{m}_i = \boldsymbol{C}_i(\boldsymbol{\kappa}_i + \boldsymbol{\Sigma}_\epsilon^{-1}(\boldsymbol{\beta}^T\boldsymbol{z}_i + \boldsymbol{\gamma}_{g_i} + s_i\boldsymbol{\alpha}^T)),$$

then sample $\boldsymbol{\psi}_i$ from

$$\boldsymbol{\psi}_i | - \sim N(\boldsymbol{m}_i, \boldsymbol{C}_i)$$

- For $i = 1, \cdots, n$ and for each $A \in \mathcal{I}$, sample the Pólya-Gamma variable $w_i(A)$ from

$$w_i(A)| - \sim \mathrm{PG}(y_i(A), \psi_i(A))$$

- Sample ϕ_α from

$$\phi_\alpha | - \sim \mathrm{Gamma}(t + \sum_{A \in \mathcal{I}} I(\alpha(A) \neq 0)/2, u + \sum_{A \in \mathcal{I}} \alpha^2(A)/2)$$

- Update $\boldsymbol{\Omega}$ with the block Gibbs sampling procedure described in Algorithm 1. For the data-augmented target distribution, set $\boldsymbol{S} = \boldsymbol{\Gamma}^T \boldsymbol{\Gamma}$.

B Runtime experiment

We conducted a runtime analysis of the Gibbs sampler for LTN for exchangeable samples. We evaluated the run time of 10 Gibbs iterations across varying number of OTUs ($K = 10, 20, 50, 100, 200, 500$) to assess computational complexity. We used the same DIABIMMUNE dataset as used in the Case Study, where number of samples $n = 777$, and we keep varying number of most abundant OTUs. We measured three timing metrics using R's system.time() function: user time (CPU time spent executing algorithm computations), system time (operating system overhead), and elapsed time (total wall-clock time). The LTN model parameters included $\lambda = 1$ and prior variance of μ is $5I$. Results (FigS1) demonstrate approximately quadratic scaling across all timing metrics, with user computation dominating ($> 99\%$) of total runtime and system overhead remaining minimal ($< 1\%$). The runtime experiments were conducted on a MacBook Air (2023) equipped with an Apple M2 chip and 16 GB unified memory.

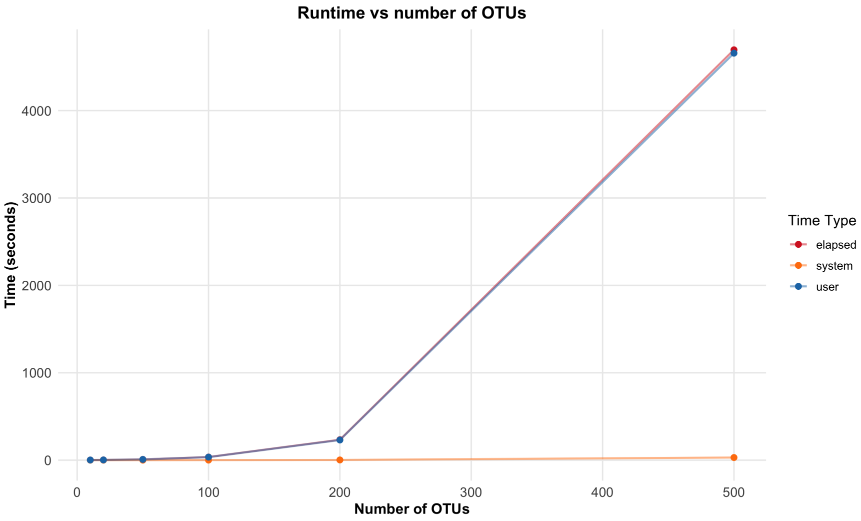

Figure S1: Runtime of 10 Gibbs iterations vs number of OTUs K

C Sensitivity analysis on tree misspecification

We investigate the robustness of the inference of mean and covariance structure to the choice of partition tree. Let $\mathcal{T}_1$ be a phylogenetic tree such that all of the right children of the internal nodes are leaves, $\mathcal{T}_2$ a balanced tree where the left and right subtree of each node have identical shape. To differentiate LTNs corresponding to different trees, we use $\mathrm{LTN}_{\mathcal{T}}$ to indicate a model constructed on tree $\mathcal{T}$. The leaves of the two trees have the same order in the pre-order traversal trace. The data $\boldsymbol{X}_{n\times K}$ is generated from $\mathrm{LTN}_{\mathcal{T}_1}(\boldsymbol{\mu}_1, \boldsymbol{\Sigma}_1)$, where number of samples $n = 200$, number of OTUs $K = 64$, $\boldsymbol{\mu}_1 = (2, \cdots, 2), \boldsymbol{\Sigma}_1 = \boldsymbol{I}$.

We fit $\mathrm{LTN}_{\mathcal{T}_1}$ and $\mathrm{LTN}_{\mathcal{T}_2}$ on $\boldsymbol{X}$ to estimate the mean and covariance of the log-odds at the internal nodes of $\mathcal{T}_1$ (ground truth) and $\mathcal{T}_2$ (misspecified tree) respectively, and convert such estimates from the misspecified tree to the correct tree $\mathcal{T}_1$.

We ran 100 replicates under this simulation setting. The MSE of $\boldsymbol{\mu}$ and marginal correlations on the original tree $\mathcal{T}_1$ averaged over the 63 internal nodes are shown in Figure S2. As expected, the estimates under the correct tree almost always have the smallest MSE. Inference under the misspecified tree seem to be more sensitive to the hyperparameter λ than the correct tree.

Figures S3 and S4 provide a closer inspection of the nodes. The estimated mean and marginal correlations of the shallow nodes are generally robust to the misspecified trees though for a small

number of node-pairs, the estimated correlation can be some-off biased. The extent of such bias is generally small for nodes in shallow levels of the tree. We emphasize that here our $\mathcal{T}_2$ is completely randomly generated. In practice, the phylogenetic and taxonomic trees are usually resembling the underlying functional tree to various extents. So the results suggest that in practice inference under LTN is generally robust to misspecification of the tree.

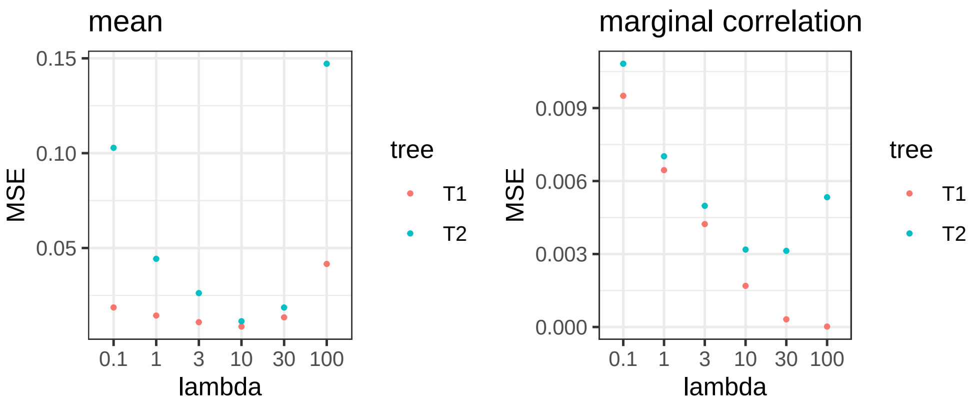

Figure S2: MSE of the estimated mean ($\boldsymbol{\mu}$) and marginal correlations on the original tree, averaged across all nodes. The MSE is calculated based on 100 replicates.

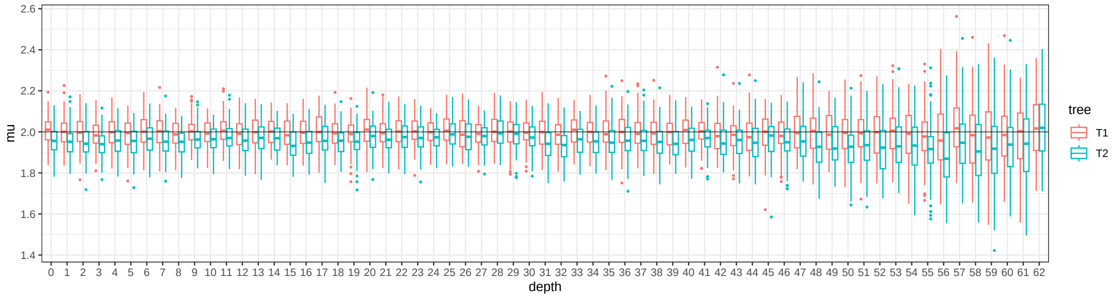

Figure S3: Estimated μ across nodes with $\lambda = 10$. The boxplots are generated based on 100 replicates. The nodes are ordered by their depth in $\mathcal{T}_1$.

The sensitivity analysis results should not be interpreted as evidence that tree choice is unimportant. Rather, our findings reveal that tree misspecification primarily affects statistical power while preserving false discovery control. When the phylogenetic tree is poorly specified, LTN's flexible covariance structure compensates by reducing sparsity in the estimated precision matrix, allowing the model to capture dependencies that the tree structure fails to represent. In contrast, under a correctly specified tree, the estimated covariance becomes nearly diagonal, reflecting the

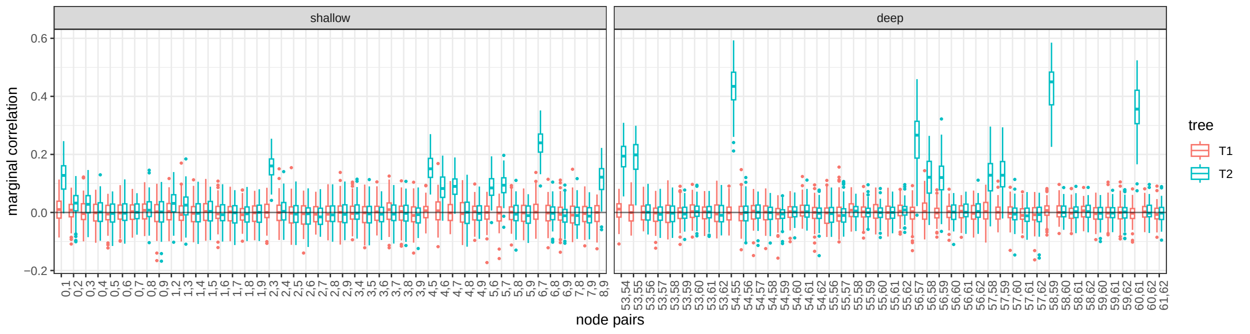

Figure S4: Estimated marginal correlations across nodes with $\lambda = 10$. The left panel shows the shallowest ten nodes in $\mathcal{T}_1$ while the right shows the deepest ten nodes. The boxplots are generated based on 100 replicates. The nodes are labeled by their depth in $\mathcal{T}_1$.

conditional independence structure imposed by the tree and leading to increased power. This adaptive mechanism distinguishes LTN from DTM, which suffers more severely from misspecification due to its rigid independence assumption that cannot compensate for incorrect tree structure. Consequently, while an appropriate tree choice is still beneficial for maximizing power, LTN's flexible covariance modeling provides robustness against false discoveries even when the tree is suboptimal.

D Details on the differential abundance analysis over dietary variables in the case study

We repeat the same abundance analysis on the microbiome compositions to each of the eight dietary factors. For each diatery variable, we treat all the other variables including the seroconversion status as covariates. Because the cessation of breastfeeding and the introduction of other types of food are registered continuously, we are essentially comparing the microbiome compositions in samples taken before and after those change points of dietary patterns. Similar to the analysis on seroconversion status, in each comparison, we include the T1D status, other dietary covariates, and environmental covariates including age, gender and nationality as fixed effects, individuals as random effects in our mixed effects model and MaAsLin2. The sample size under different comparisons and the PJAPs (with $m = 1 - 0.5^{1/99}$) are shown in Table S1.

Table S1: PJAPs of the dietary variables

Variable	Group size		PJAP
	On ($s_i = 1$)	Off ($s_i = 0$)	
Barley	531	246	1.00
Breastfeeding	248	529	1.00
Buckwheat&Millet	198	579	1.00
Eggs	477	300	0.75
Fish	581	196	1.00
Rye	514	263	0.97
Solid Food	681	96	1.00
Soy Product	107	670	1.00

The PMAPs (computed with $m = 0.05$) are again visualized on the phylogenetic tree along with the significant OTUs reported under MaAsLin2 with BH-adjusted q-values for solid food (Figure S5), soy products (Figure S6) and breastfeeding (Figure S7). The PMAP plots for the other dietary variables are reported in Figures S8 - S12).

Two distinct features of the tree-based parametrization are demonstrated in these figures. First, our model appears to lead to a sparser representation of the signals than the OTU-level analysis when cross-group differences are detected at multiple OTUs that are close to each other on the tree. For example, as shown in Figure S5, significant associations with solid food have been identified by our LTN-based mixed-effects model at relatively shallow nodes on the phylogenetic tree. In contrast, MaAsLin2 reported very large sets of significant descendant OTUs of these nodes. More interestingly, there is a node with high PMAP whose left and right child are Firmicutes and Bacteroidetes respectively (see Figure S5), indicating that introduction of solid food is associated with changes in the "Firmicutes/Bacteroides ratio", which is widely used as an index of dysbiosis (Stojanov et al., 2020). Second, "chains" of high PMAPs are observed in several comparisons, which can be useful in targeting the more precise taxa contributing to the cross-group difference. For example, in Figure S6 of the posterior marginal alternative probabilities of soy product, a chain of three ancestors of OTU 4439360 implies higher relative abundance of OTU 4439360 after introducing soy product, which is consistent with data at older age. The switching signs of $\hat{\alpha}$ at the three nodes in the chain $(+, -, +)$ indicate increase in relative abundance of the left, right, and left

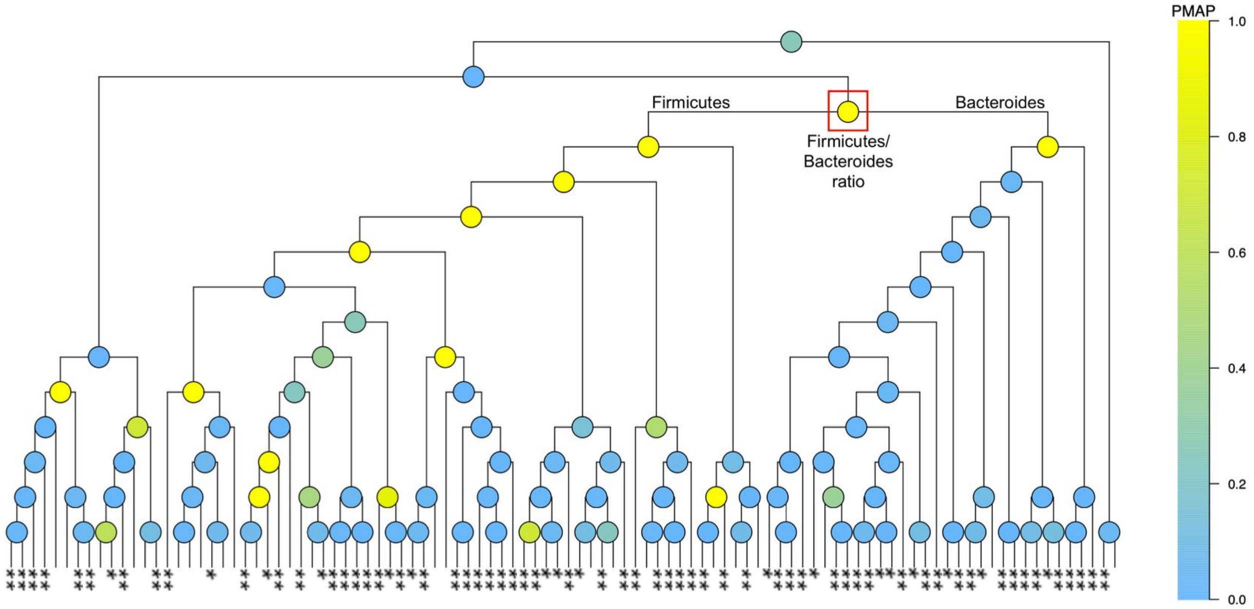

Figure S5: PMAPs of the introduction of solid food. The significant OTUs reported by MaAsLin2 are marked in the same way as in the seroconversion PMAP figure in the main text.

branches respectively.

Finally, we investigate how breastfeeding is associated with the gut microbiome development. In the comparison between samples collected before and after cessation of breastfeeding, the posterior marginal alternative probabilities are visualized in Figure S7, and for each significant node A, the taxa associated with the left and right child of this node, the common taxon they belong to, as well as the posterior mean $\hat{\alpha}(A)$, are summarized in Table S2. The main discovery on breastfeeding in the original study by Kostic et al. (2015) is an increase in Bifidobacterium and Lactobacillus species and reduction in Lachnospiraceae during breastfeeding. Table S2 and Figure S7 reveals some similar findings and more. The negative $\hat{\alpha}$ at node 7 indicates reduced Lachnospiraceae relative to Veillonellaceae in samples collected from infants during breastfeeding. Moreover, Bifidobacterium has higher relative abundance in the samples collected during breastfeeding period; indeed, the positive $\hat{\alpha}$ at nodes 4 and 5 (and their ancestors) indicates that such enrichment of Bifidobacterium can be attributed to longum and bifidum as well as some other unclassified species. Such result is consistent with previous findings on the high abundance of Bifidobacterium longum

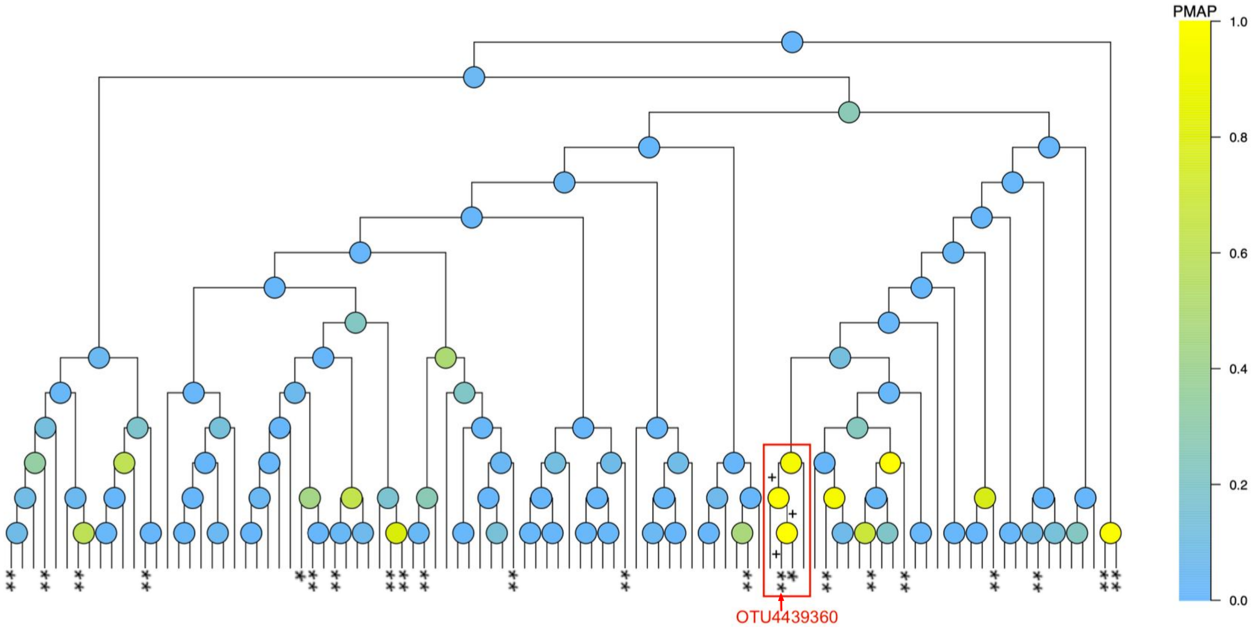

Figure S6: PMAPs of the introduction of soy products. The chain of three significant nodes and their descendant OTU 4439360 are marked in the figure. The posterior mean of α at these three nodes (from top to bottom) are 1.22, -3.12, and 3.19 respectively. The significant nodes reported by our LTN-based mixed-effects model and the significant OTUs reported by MaAsLin2 are marked in the same way as in the seroconversion PMAP figure in the main text.

in breastmilk and enrichment of Bifidobacterium bifidum in breastfed infants (Gueimonde et al., 2007; Fehr et al., 2020).

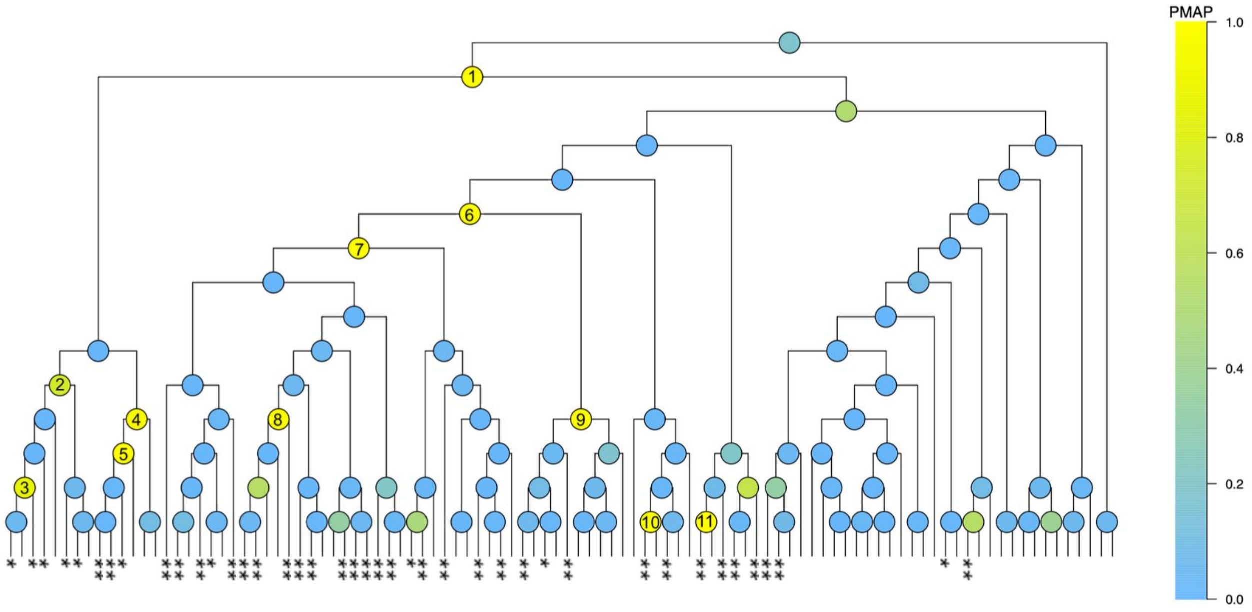

Figure S7: PMAPs of cessation of breastfeeding. The significant OTUs reported by MaAsLin2 are marked in the same way as in the seroconversion PMAP figure in the main text.

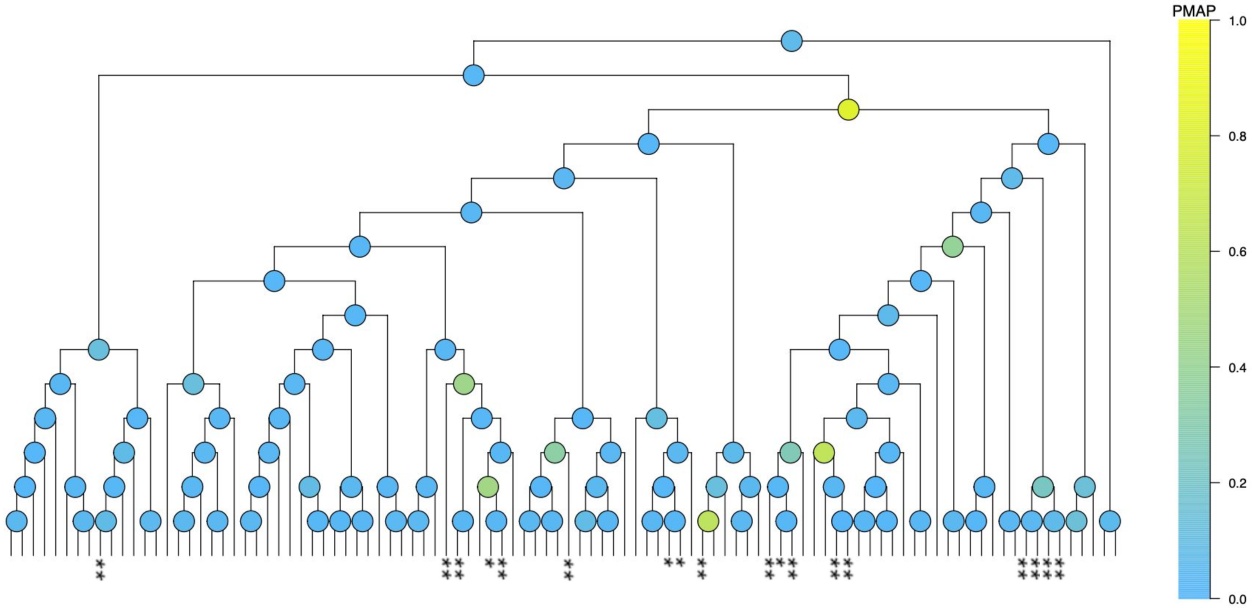

Figure S8: PMAPs for rye ($m = 0.05$)

Table S2: Nodes with significant association with breastfeeding reported by our LTN-based mixed-effects model with posterior expected FDR ≤ 0.05. The node labels are the same as in Figure S7.

node	taxon in common	taxa on left	taxa on right	posterior mean $\hat{\alpha}$
1	Bacteria	Proteobacteria, Actinobacteria	Firmicutes, Bacteroidetes	0.52
2	Proteobacteria	Gammaproteobacteria	Betaproteobacteria	0.94
3	Enterobacteriaceae	OTU 782953, OTU 668514	OTU 2119418	1.28
4	Actinobacteria	Actinobacteria	Coriobacteriia	0.97
5	Bifidobacterium	longum, bifidum, unclassified	adolescentis	1.41
6	Clostridiales	Lachnospiraceae, Veillonellaceae	Ruminococcaceae	1.11
7	Clostridiales	Lachnospiraceae	Veillonellaceae	-1.61
8	Lachnospiraceae	OTU 289734, OTU 4483337, OTU 2724175, OTU 4448492	OTU 4469576	1.35
9	Ruminococcaceae	Oscillospira, Faecalibacterium, Ruminococcus	unclassified	-1.29
10	Clostridiaceae	OTU 193672	OTU 3576174	-2.24
11	Streptococcus	OTU 4442130	OTU 4425214	1.50

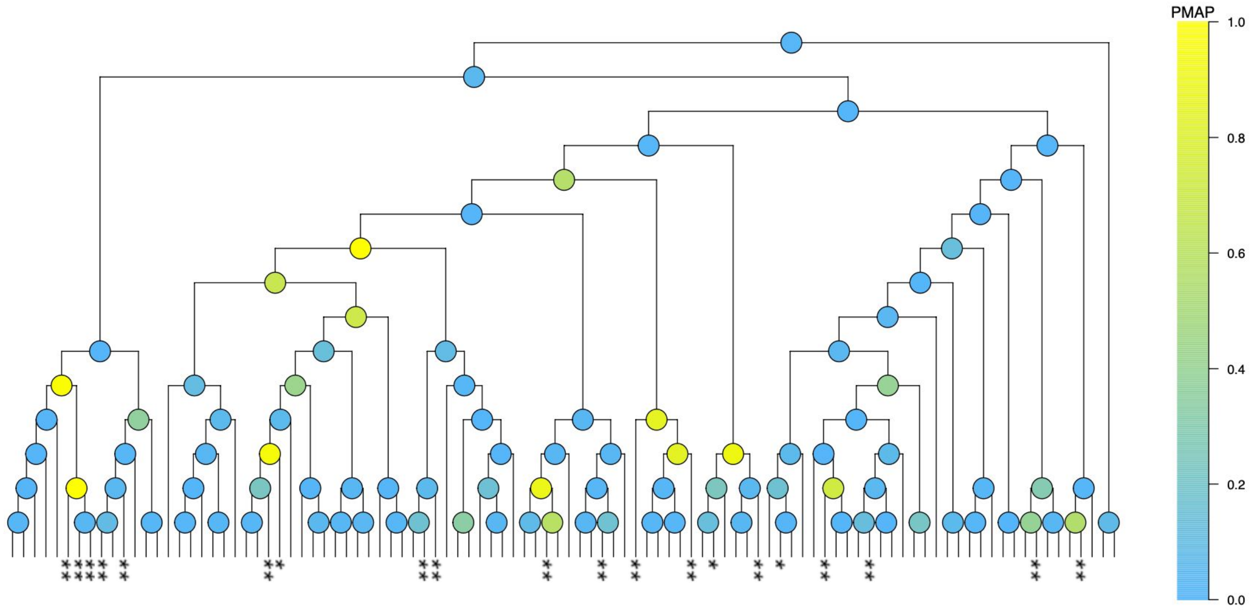

Figure S9: PMAPs for Buckwheat and Millet ($m = 0.05$)

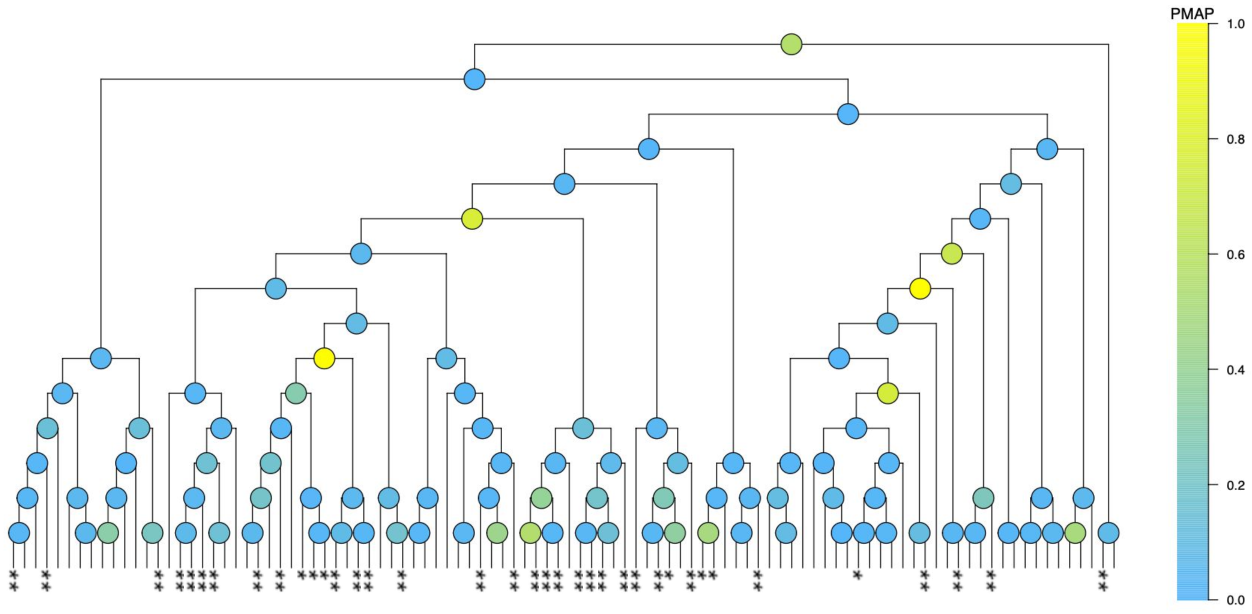

Figure S10: PMAPs for eggs ($m = 0.05$)

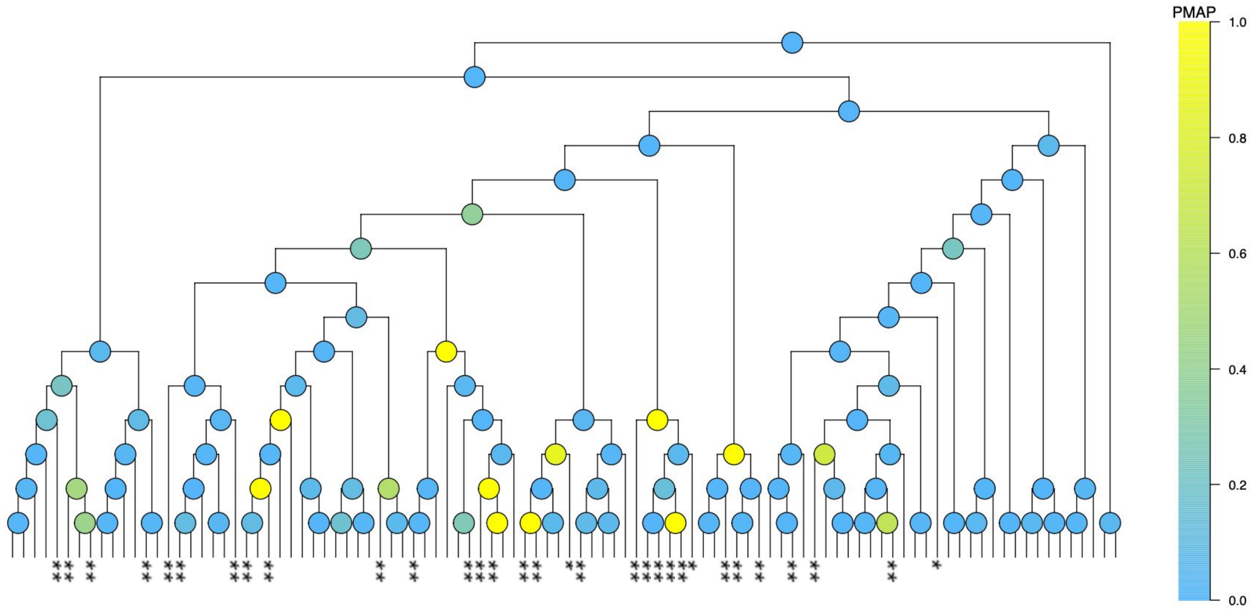

Figure S11: PMAPs for fish ($m = 0.05$)

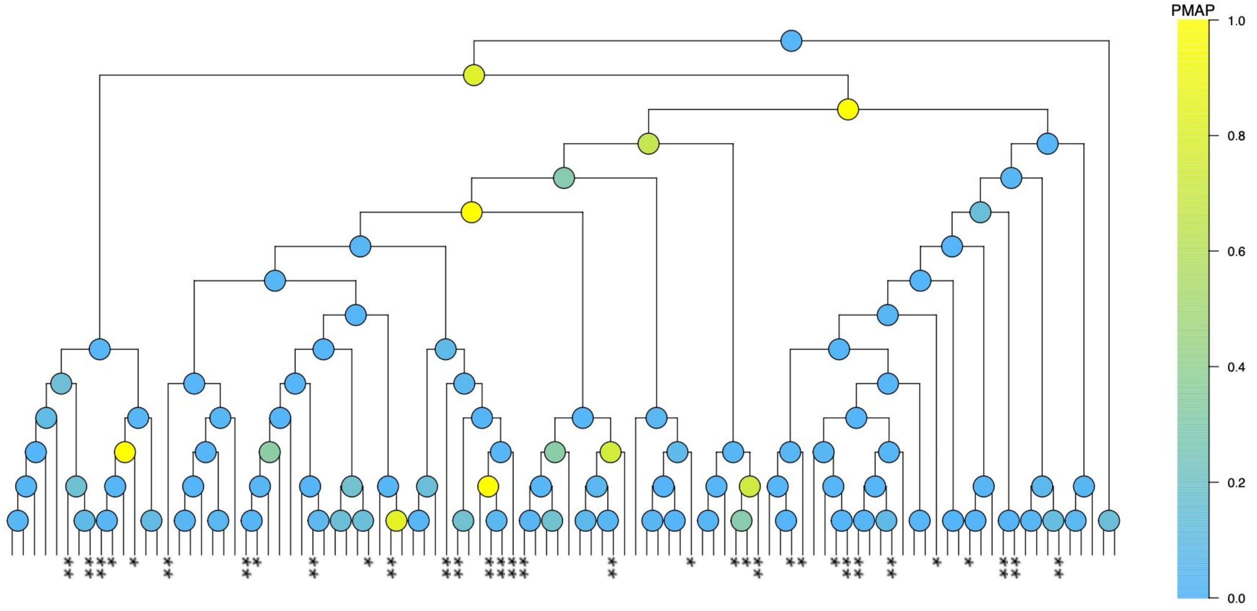

Figure S12: PMAPs for barley ($m = 0.05$)

References

Fehr, K., S. Moossavi, H. Sbihi, R. C. T. Boutin, L. Bode, B. Robertson, C. Yonemitsu, C. J. Field, A. B. Becker, P. J. Mandhane, M. R. Sears, E. Khafipour, T. J. Moraes, P. Subbarao, B. B. Finlay, S. E. Turvey, and M. B. Azad (2020, Aug). Breastmilk feeding practices are associated with the co-occurrence of bacteria in mothers' milk and the infant gut: the child cohort study. *Cell Host Microbe 28*(2), 285–297.

Gueimonde, M., K. Laitinen, S. Salminen, and E. Isolauri (2007). Breast milk: a source of bifidobacteria for infant gut development and maturation? *Neonatology 92*(1), 64–66.

Kostic, A. D., D. Gevers, H. Siljander, T. Vatanen, T. Hyötyläinen, A.-M. Hämäläinen, A. Peet, V. Tillmann, P. Pöhö, I. Mattila, H. Lähdesmäki, E. A. Franzosa, O. Vaarala, M. de Goffau, H. Harmsen, J. Ilonen, S. M. Virtanen, C. B. Clish, M. Orešič, C. Huttenhower, M. Knip, and R. J. Xavier (2015, 2020/12/18). The dynamics of the human infant gut microbiome in development and in progression toward type 1 diabetes. *Cell Host & Microbe 17*(2), 260–273.

Polson, N. G., J. G. Scott, and J. Windle (2013). Bayesian inference for logistic models using pólya–gamma latent variables. *Journal of the American Statistical Association 108*(504), 1339–1349.

Stojanov, S., A. Berlec, and B. Štrukelj (2020, 11). The influence of probiotics on the firmicutes/bacteroidetes ratio in the treatment of obesity and inflammatory bowel disease. *Microorganisms 8*(11), 1715.
